# Pharmacology, Physiology and Genetics of the Neuropeptide S System

**DOI:** 10.3390/ph14050401

**Published:** 2021-04-23

**Authors:** Rainer K. Reinscheid, Chiara Ruzza

**Affiliations:** 1 Institute of Pharmacology & Toxicology, University Hospital Jena, Friedrich-Schiller University, 07747 Jena, Germany; 2 Institute of Physiology I, University Hospital Münster, Westfälische-Wilhelms University, 48149 Münster, Germany; 3 Department of Neuroscience and Rehabilitation and Laboratory for Technologies of Advanced Therapies (LTTA), University of Ferrara, 44121 Ferrara, Italy

**Keywords:** transmitter, GPCR, brainstem, anxiety, memory, genetic variation

## Abstract

The Neuropeptide S (NPS) system is a rather ‘young’ transmitter system that was discovered and functionally described less than 20 years ago. This review highlights the progress that has been made in elucidating its pharmacology, anatomical distribution, and functional involvement in a variety of physiological effects, including behavior and immune functions. Early on, genetic variations of the human NPS receptor (NPSR1) have attracted attention and we summarize current hypotheses of genetic linkage with disease and human behaviors. Finally, we review the therapeutic potential of future drugs modulating NPS signaling. This review serves as an introduction to the broad collection of original research papers and reviews from experts in the field that are presented in this Special Issue.

## 1. NPS and the Orphan Receptor Strategy

**Figure 1:**
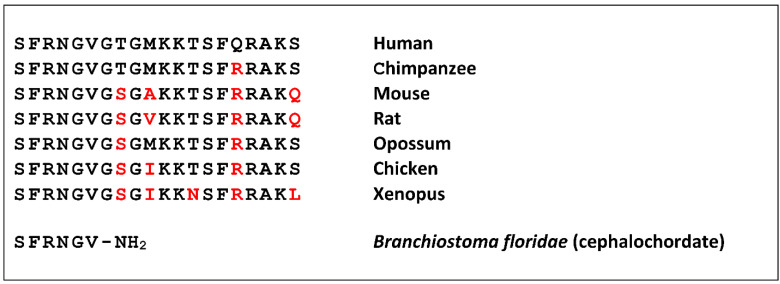
Neuropeptide S (NPS) or NPS-like peptide sequences. Residues divergent from the human NPS sequence are highlighted in red. The C-terminal amide (NH_2_) in the Branchiostoma peptide is encoded by glycine in the precursor protein and therefore identical to the consensus.

Neuropeptide S (NPS) was discovered as a ligand of a previously orphan G protein-coupled receptor (GPCR) by using the “orphan receptor strategy”, also known as “reverse pharmacology” [1]. The receptor (previously known as GPR154 or GPRA) was stably expressed in cells that served as a bait to purify the endogenous ligand from brain extracts [2]. The ligand turned out to be a peptide of 20 amino acids that contains a perfectly conserved serine (single amino acid code “S”) at the N-terminus in all species analyzed, and was thus termed accordingly [3]. NPS is encoded as a single copy peptide by a rather small precursor protein (<90 amino acids) that occurs in the genome of all tetrapods but is absent from fish [4]. The seven N-terminal residues are identical in all tetrapods, as well as the overall 20 amino acid length, while the C-terminal half shows more variation (Figure 1).

A shortened peptide has been identified in a cephalochordate that contains the conserved amino terminal residues [5]. Together with the identification of a distantly-related GPCR from the same class of animals, this suggests a rather complex evolution of the NPS system in bilaterians, where teleost fish may have lost both genes [6].

NPSR1 is also a single-copy gene with moderate similarity to other peptide GPCRs, the closest being vasopressin 1A and oxytocin receptors. NPSR1 is found in the genomes of all tetrapods and no convincing orthologues have been identified in fish genomes [7].

## 2. General Pharmacology

NPSR1 couples via Gα_s_ and Gα_q_ to elevate intracellular cAMP and Ca^2+^, thus it is an excitatory GPCR [8]. Activation of mitogen-activated protein kinase (MAPK) pathways and opening of neuronal Ca^2+^ channels have also been described [8,9,10]. NPS activates its receptor at low nanomolar concentrations and structure-activity relationship (SAR) studies have confirmed the importance of the amino terminus for agonist activity, overlapping with the high evolutionary conservation of this part of the peptide structure [11,12,13]. Focused SAR studies performed on Gly^5^ lead to the identification of peptidergic NPSR1 antagonists, characterized by a D-amino acid with a short branched aliphatic side chain [14,15]. Some well-characterized NPSR1 antagonists identified in the frame of these studies are [D-Cys(*t*-Bu)^5^]NPS, [D-Val^5^]NPS, and [*t*-Bu-D-Gly^5^]NPS [15,16,17]. 

**Figure 2:**
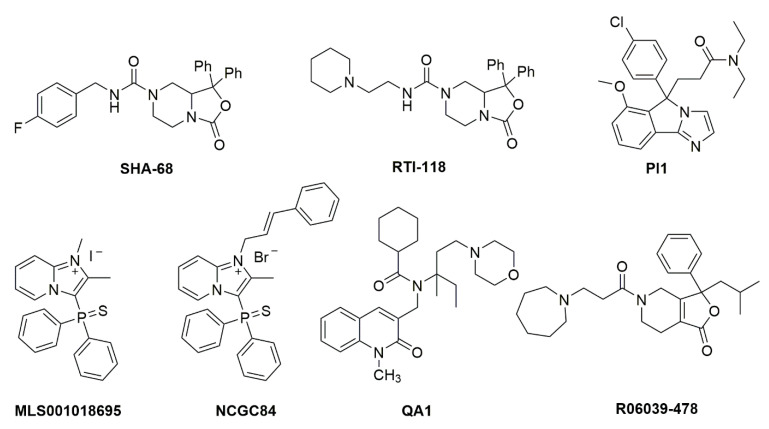
Chemical structures of NPSR1 antagonists. Details about synthesis and pharmacological activities can be found in the original literature. SHA 68 [18], RTI-118 [68], PI1 [118], MLS001018695 [119], NCGC84 [23], QA1 [120], R06039-478 [121].

The prototype synthetic NPSR1 antagonist is SHA 68, belonging to the class of diphenyltetrahydro-1*H*-oxazolo [3,4-α]pyrazines [18] (Figure 2). SHA 68, and the closely related SHA 66, display nanomolar affinity for NPSR1 in vitro but only limited bioavailability in vivo, due to its high lipophilicity. Using the core structure of SHA 68, several refinements have been made to improve in vivo potency. It appears that slight increases in polarity can indeed cause an increased in vivo potency, albeit with a net loss in receptor affinity [19,20,21,22]. Additional high-throughput drug screening programs have yielded further structures with NPSR1 antagonistic profiles (Figure 2), although with lower in vitro and in vivo potency compared to SHA 68 [23,24,25]. None of these compounds has progressed beyond the preclinical stage yet.

## 3. Anatomical Distribution of the NPS System

Expression of NPS precursor and receptor mRNA have been studied in detail in brain. In rats and mice, the NPS precursor mRNA is expressed in only a few brainstem nuclei, most notably the pericoerulear region and the lateral parabrachial area including the Kölliker–Fuse nucleus [3,26,27]. In rat brain, additional NPS expression is detected in the principal sensory trigeminal nucleus and a few scattered cells in hypothalamus and amygdala. Widespread co-localization with excitatory neurotransmitters—including glutamate, acetylcholine, CRF or galanin—in the brainstem nuclei supports the view that NPS is part of an excitatory signaling system. Notably, in rat or mouse locus coeruleus (LC) NPS is not colocalized with noradrenaline in the LC proper, but found in immediately adjacent neurons. Using transgenic NPS-EGFP mice, the total number of NPS-expressing neurons in the mouse brain was determined to be ~500 [28], which makes it one of the rarest neuropeptides in the brain. Immunohistochemical analyses, however, revealed widespread projections of NPS-immunopositive fibers throughout the mouse brain, with highest densities found in hypothalamus, thalamus, and structures of the extended amygdala [27,28]. It is currently unkown if these fiber projections also represent synaptic terminals, but a few studies have investigated in vivo NPS release in certain brain areas. For example, increased NPS release upon stress exposure has been detected in the rat amygdala by microdialysis, demonstrating that it is a functional neurotransmitter [29]. Regulation of NPS transcripts has been observed in rat brain after sleep deprivation [30] or treatment with neuroleptics [31,32]. 

NPSR1 mRNA expression is found in many areas of the rodent brain, with highest densities in several cortical structures, thalamic nuclei, hypothalamus, subiculum, and amygdala [3,26,27]. Neuroleptics and chronic morphine also appear to regulate NPSR1 mRNA in certain areas of the rat brain [31,32,33].

Expression of NPS precursor or receptor mRNA in peripheral tissues has not been studied by in-situ hybridization but bulk mRNA has been quantified from whole organs. In rats, precursor and receptor mRNA was detected by RT-PCR in endocrine tissues, including mammary and salivary glands as well as thyroid [3]. Endogenous expression of NPSR1 was also observed in neuroendocrine tumors [9] and established colon cancer cell lines [8]. 

Information about NPS expression in the human brain is limited as only the pons has been investigated in detail. Similar to rodents, clusters of NPS-expressing neurons were found in the human lateral parabrachial nucleus as well as the pericoerulear region, although with a different quantitative distribution pattern compared to rodents [34]. An additional cluster of NPS mRNA-positive neurons was identified in the pontine central gray matter in both human and rat brain. The overall estimate for the human pons predicts a number of ~22,000 NPS-producing neurons. NPSR1 expression was detected by in-situ hybridization in various human tegmental nuclei and the periaqueductal gray, however, a comprehensive brain map for human or primate NPSR1 has yet to be produced. 

Anatomical studies using anti-NPSR1 antibodies have so far not been verified by using knockout controls to validate the specificity of the antisera [9,35,36,37,38]. Therefore, the precise subcellular location of the receptor proteins is currently unknown. 

## 4. Physiological and Behavioral Effects of NPS

### 4.1. Modulation of Animal Behavior

In experimental animals, NPS was found to induce arousal and wakefulness [3,39], reduce fear and anxiety [3,39,40,41,42,43,44], promote learning and memory consolidation [45,46,47], accelerate fear extinction [48,49], attenuate pharmacologically-induced psychotic behaviors [50], stimulate release of stress hormones [51,52] and prefrontal dopamine [53], produce analgesia [54,55,56,57,58,59], attenuate food intake [51,60,61], counteract motor deficits in a model of Parkinson’s disease [62], and promote drug-seeking behaviors including relapse of drug seeking [63,64,65,66,67]. An overview about animal models, routes and location of drug administration, and behavioral paradigms can be found in the review by Ruzza et al. [25]. Importantly, NPS-dependent stimulation of, or relapse to, either alcohol or cocaine seeking behavior can be blocked by NPSR1 antagonists [67,68]. Hypothalamic NPSR1 appear to be critical for modulating drug seeking behaviors and may interact with orexin/hypocretin and corticotropin-releasing factor neurotransmission [63,69,70]. Notably, both central (intracerebroventricular) and intra-amygdala administration of NPS elicited acute anxiolytic-like effects [3,48], while intra-amygdala administration of SHA 68 produced increased anxiety-like behavior [48], suggesting endogenous NPS release upon stress exposure. It appears that NPS may have a unique bifurcated effect on anxiety: while acutely attenuating anxiety-like responses, it later appears to facilitate extinction of fear memories. This combination of activities could be desirable therapeutic effects in the treatment of generalized anxiety disorders, phobias, panic disorder, or post-traumatic stress disorder.

There is limited evidence for a role of NPS in mood regulation. In the high-anxiety Flinders rat model, NPS normalized anxiety-like behaviors without producing anti-depressant effects in the forced swim test [71]. However, localized NPS administrations into the nucleus accumbens (NAc) shell, but not the NAc core or the bed nucleus of the stria terminalis (BNST), were reported to induce anti-depressant effects in a learned helplessness model in rats [72]. Unexpectedly, infusions of the NPSR1 antagonist SHA 68 into the BNST, another important part of the extended amygdala network regulating mood and anxiety, also produced anti-depressant-like effects. However, knockout mouse models did not exhibit depression-like phenotypes. Further studies may be required to clarify possible functions of endogenous NPS in mood regulation.

Prominent phenotypes in knockout mouse models for NPSR1 and NPS include attenuated arousal, deficits in learning and memory including disrupted fear learning, and mildly increased anxiety [45,52,73,74,75]. While the anxiogenic and memory impairment phenotype of NPSR1 knockout mice has not been replicated in all laboratories [76], all studies showed that the stimulant, arousal-promoting, and anxiolytic effects of NPS completely disappeared in NPSR1 knockout animals, demonstrating that NPSR1 is the unique protein by which NPS exerts its biological actions [52,73,74,76]. 

No respiratory phenotypes were detected in NPSR1 knockout mice, including models of induced asthma [77]. However, central NPS administration can stimulate respiratory frequency in an NPSR1-dependent manner [78].

### 4.2. NPS and Immune Functions

A few in vitro studies have described effects of NPS on lymphocytes or macrophages [79,80,81]. In general, NPS was found to induce lymphocyte proliferation and promote macrophage phagocytosis. These effects were accompanied by upregulated expression of pro-inflammatory cytokines. Whether any immune-modulatory deficits exist in NPSR1 or NPS knockout mice has not yet been established. Increased expression of NPSR1 in eosinophils from patients with asthma or severe inflammation has been reported [82]. 

## 5. Genetics of the Human NPS System and Associations with Disease and Behavior

The region on human chromosome 7 harboring NPSR1 was first identified in a positional cloning approach to contain an asthma-susceptibility gene. NPSR1 was one of the candidate genes and was termed GPRA (G protein-coupled receptor for asthma susceptibility) [35]. Further studies have reported genetic linkage of the NPSR1 locus on human Chr7 with inflammatory or allergic disorders, including asthma, airway hyper-responsiveness, atopic dermatitis, inflammatory bowel syndrome, and rheumatoid arthritis [83,84,85,86,87,88,89,90,91,92,93,94], although some studies failed to replicate such associations [95,96,97,98]. Complex haplotypes in and surrounding the NPSR1 gene, rather than individual single nucleotide polymorphisms (SNPs), were identified in these association studies and a detailed physiological mechanism has not yet been described to explain NPSR1-related functions in allergic or inflammatory diseases. Furthermore, NPSR1 knockout mice did not display asthma-related phenotypes or deficits in respiratory functions [77]. Instead, a neurogenic mechanism involving brainstem NPSR1 has been suggested to regulate NPS-dependent respiratory effects in mice [78]. Despite strong human genetic data, the physiological role of NPSR1 in asthma, allergies, or inflammation remains unclear.

Phenotypes of a missense mutation in NPSR1 received considerable attention because the Ile^107^Asn variants (rs324981, T/A) display 5–10 fold differences in agonist potency while binding affinity is not affected [8,99]. The rs324981 SNP is human-specific and the derived A-allele produces NPSR1-Asn^107^ with significantly reduced receptor expression and attenuated functionality. The NPRS1-Asn^107^ variant is also part of several haplotypes that have been associated with allergic and inflammatory diseases and was postulated to provide some protective effect, despite not showing any significant association on its own. 

Due to its pharmacological phenotype, rs324981 was studied most intensely in humans and has been associated with panic disorder, anxiety, fear evaluation, stress responsiveness, maladaptive personality traits, early-onset bipolar disorder, and sleep [100,101,102,103,104,105,106,107,108,109,110,111]. Paradoxically, the ancestral T-allele, coding for the high-sensitivity variant NPSR1-Ile^107^, was often associated with disease phenotypes, except for a schizophrenia study where T-carriers displayed improved cognitive performance [112]. Tests in healthy human volunteers revealed T-carrier-specific associations with over-interpretation of fear or fearful stimuli, and functional brain scans showed rs324981 allele-dependent changes in neuronal activity in discrete brain areas in controls vs. panic or anxiety disorder patients, in particular involving specific subdivisions of the prefrontal and orbitofrontal cortex [104,106,107,109,113].

Surprisingly, the derived A-allele of rs324981 (encoding the low-functionality variant NPSR1-Asn^107^) has become the major allele in European and Asian populations, suggesting some form of selection. Analysis of worldwide rs324981 allele frequencies suggests an African origin of the mutation before the onset of migrations by anatomically-modern humans, consistent with the out-of-Africa hypothesis [114].

Recently, a gain-of-function variant (NPSR1-His^206^) has been described in a single family that dramatically reduces total sleep time [115]. This observation confirms earlier genetic association reports of the high-activity variant NPSR1-Ile^107^ with delayed bedtime [101] and supports a significant role for NPS in arousal. 

A mutation reducing agonist potency 10–20 fold has been identified in the NPS peptide itself, producing Leu^6^-NPS (instead of Val^6^; rs4751440, G/C) [94,116]. Thus far, no phenotypes have been associated with this variant, which might be due to the relatively low allele frequency and geographically restricted occurrence in European and Indian populations. Interestingly, genetic analysis showed that the rs4751440 mutation may have originated in Neandertals, since it is found in all currently available Neandertal genomes. Most likely, the mutant allele was introduced into the anatomically-modern human gene pool by interbreeding with Neandertals in Southern Europe after the main migrations to East Asia had already occurred [114].

## 6. Therapeutic Potential

The prospect of a non-sedative anxiolytic stimulated a lot of interest in the pharmaceutical industry to search for small-molecule NPSR1 agonists. However, so far, no such molecules have been discovered. In contrast, various independent structures of small-molecule NPSR1 antagonists have been described (Figure 2), but thus far none of them have entered clinical trials. Based on preclinical data, such compounds may have therapeutic potential as sleep medications or in the treatment of drug addiction, especially to prevent relapse of drug abuse [23,25,68,117].

## 7. Concluding Remarks

An impressive number of physiological functions have been identified for the NPS system in the relatively short time since its discovery. Together with the plethora of genetic association data for NPSR1 variants with human disease and behaviors, increased efforts to identify therapeutic applications for this interesting transmitter system appear to be promising and warranted. The articles in this Special Issue reflect the continuing progress in our knowledge about the NPS system and its therapeutic potential.

## Data Availability

Data sharing not applicable.
